# The crosstalk between alternative splicing and circular RNA in cancer: pathogenic insights and therapeutic implications

**DOI:** 10.1186/s11658-024-00662-x

**Published:** 2024-11-16

**Authors:** Hongkun Hu, Jinxin Tang, Hua Wang, Xiaoning Guo, Chao Tu, Zhihong Li

**Affiliations:** 1 Department of Orthopaedics, Hunan Key Laboratory of Tumor Models and Individualized Medicine, Hunan Engineering Research Center of Artificial Intelligence-Based Medical Equipment, The Second Xiangya Hospital of Central South University, Changsha, 410011 China; 2 Hunan Key Laboratory of Tumor Models and Individualized Medicine, The Second Xiangya Hospital of Central South University, Changsha, 410011 China; 3 Hunan Engineering Research Center of Artificial Intelligence-Based Medical Equipment, The Second Xiangya Hospital of Central South University, Changsha, 410011 Hunan China

**Keywords:** Alternative splicing, Circular RNA, Histone modification, N^6^-methyladenosine modification, Splicing factor

## Abstract

RNA splicing is a fundamental step of gene expression. While constitutive splicing removes introns and joins exons unbiasedly, alternative splicing (AS) selectively determines the assembly of exons and introns to generate RNA variants corresponding to the same transcript. The biogenesis of circular RNAs (circRNAs) is inextricably associated with AS. Back-splicing, the biogenic process of circRNA, is a special form of AS. In cancer, both AS and circRNA deviate from the original track. In the present review, we delve into the intricate interplay between AS and circRNAs in the context of cancer. The relationship between AS and circRNAs is intricate, where AS modulates the biogenesis of circRNAs and circRNAs in return regulate AS events. Beyond that, epigenetic and posttranscriptional modifications concurrently regulate AS and circRNAs. On the basis of this modality, we summarize current knowledge on how splicing factors and other RNA binding proteins regulate circRNA biogenesis, and how circRNAs interact with splicing factors to influence AS events. Specifically, the feedback loop regulation between circRNAs and AS events contributes greatly to oncogenesis and cancer progression. In summary, resolving the crosstalk between AS and circRNA will not only provide better insight into cancer biology but also provoke novel strategies to combat cancer.

**Graphical Abstract:**

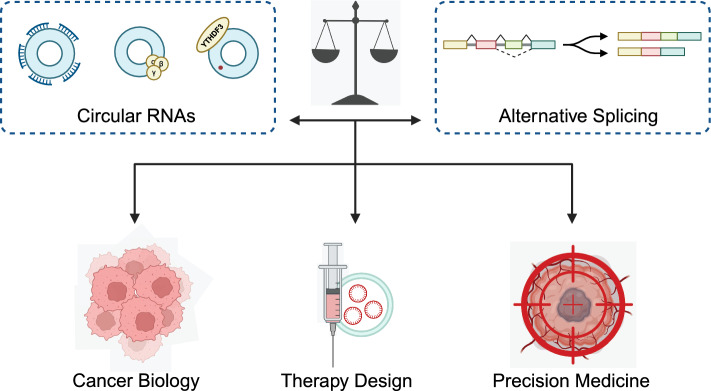

## Introduction

RNA splicing is a fundamental step of gene expression that involves the editing of pre-messenger RNA (pre-mRNA) transcripts to remove non-coding sequences, known as introns, and join coding sequences, called exons. Apart from constitutive splicing which removes introns and assembles remaining exons unbiasedly, alternative splicing (AS) selectively determines exon inclusion or exclusion, engendering multiple mRNA isoforms according to one pre-mRNA. AS is more a rule than an exception as it takes place in about 95% of human genes and gives rise to phenotypic diversity [1, 2]. In cancer, dysregulation of RNA splicing and aberrant AS have emerged as critical factors in tumorigenesis and contribute to the hallmarks of cancer, leading to tumor progression, relapse, and therapy resistance [3, 4].

Apart from linear mRNA transcripts, the biogenesis of circular RNAs (circRNAs) is also inextricably associated with AS. CircRNAs are a class of non-coding RNAs (ncRNAs) characterized by their covalently closed loop structure, which confers stability and resistance to exonuclease degradation. In cancer, circRNAs have been identified as key regulators of gene expression and dysregulation of circRNA expression profiles has been implicated in oncogenesis, metastasis, and therapy resistance across various cancer types [5]. The multifaceted roles of circRNAs in cancer highlight their potential as biomarkers for diagnosis and prognosis, as well as therapeutic targets [6, 7]. The relationship between AS and circRNAs is intricate, as circRNAs themselves act as regulators of AS events by modulating the splicing machinery while AS imposes significant influence on circRNA biogenesis [8]. Consequently, the dynamic interplay between AS and circRNA has profound implications for gene expression and is associated with numerous physiological processes and diseases, including cancer [9]. Moreover, recent advances have revealed that epigenetic and pre-mRNA modification concurrently regulates AS and circRNA biogenesis, which adds to the complexity of this ingenious regulatory network.

In the present review, we delve into the intricate interplay between AS and circRNAs in the context of cancer. By depicting the interaction between circRNAs and the splicing machinery, we elaborate on how AS events dictate the formation of circRNAs, and how these circRNAs, in turn, regulate AS patterns. We also point out epigenetic and posttranscriptional modifications that hinge on the crosstalk between AS and circRNAs. As we navigate through this complex web of interactions, we hope to provide a comprehensive understanding that could pave the way for innovative therapeutic strategies in the fight against cancer.

## Alternative splicing

### Principle of alternative splicing

**Fig. 1:**
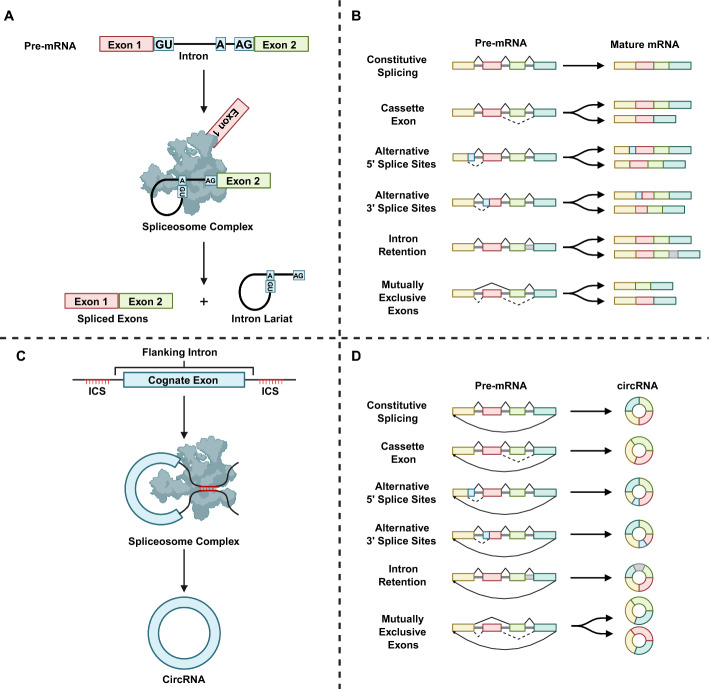
Alternative splicing and alternative back-splicing. Linear splicing (**A**) of pre-mRNA removes introns and joins exons to produce mRNA. While constitutive splicing arranges exons and introns unbiasedly, alternative splicing (**B**) selectively assembles exons and introns to engender multiple mRNA variants corresponding to the same pre-mRNA. Back-splicing (**C**) shares the same splicing machinery with linear splicing and relies on the inverted complementary sequences (ICSs) in the flanking introns. Similarly, alternative back splicing (**D**) produces circRNA variants. CircRNA, circular RNA; mRNA, messenger RNA

Splicing of pre-mRNAs is carried out by a megadalton protein complex termed spliceosome (Fig. 1A). Spliceosome components, including small nuclear ribonucleoproteins (snRNPs) and associated proteins, act sequentially on specific splicing sites and catalyze the removal of the introns, covalently joining the adjacent exons [10–12]. Splicing events are determined by the affinity of the splicing site to the spliceosome. Splicing sites with stronger affinity more efficiently recruit spliceosome components and typically lead to constitutive splicing, the generation of dominant mRNA products. AS occurs when “weaker” splicing sites are used for a splicing event, generating mRNA variants.

AS is regulated by both cis-acting motifs within the pre-RNA and trans-acting RNA binding proteins termed splicing factors by attuning the binding affinity between splicing sites and spliceosome. Cis-acting motifs are located in both introns and exons. While most of them serve as binding sites for splicing factors, some sequences also function as complementary elements to influence the spatial structure of pre-mRNAs, thereby regulating AS [13–15]. Splicing factors elicit pleiotropic effects on different pre-mRNA transcripts. Although splicing factors of the same family may share certain characteristics, such as heterogeneous nuclear ribonucleoprotein particle (hnRNP) proteins that generally inhibit splicing, the exact effect of splicing factors on an AS event is highly context-specific [16]. Additionally, as RNA binding proteins, splicing factors are also involved in the regulation of other genetic behaviors, such as transcription, nuclear export, and translation-dependent mRNA decay [17].

On the basis of the final structure of mRNA products, AS events can be categorized into five distinct modes (Fig. 1B). Functional AS events lead to the expression of mRNA and protein isoforms. However, in some instances AS events, especially intron retention, result in the introduction of premature stop codons and subsequently cause nonsense-mediated decay (NMD) of the mRNA or production of a truncated protein [18].

### Alternative splicing in oncogenesis

Multiple studies have confirmed that alteration in global AS pattern is involved in oncogenesis. Pan-cancer analysis showed that AS events occur up to 30% more frequently in cancerous tissues than normal samples [19]. Hot-spot genes that are alternatively spliced across multiple cancer types are generally associated with fundamental hallmarks of tumor progression such as apoptosis resistance [20]. This includes both the upregulation of oncogenes and the loss of tumor suppressive genes [21, 22]. Additionally, cancer-specific AS events create new exon-exon junctions (also known as neojunctions), which contribute to the generation of neoantigens. AS-derived neoantigens are tumor-specific and likely conservative across cancer types, implying a great potential as targets of immunotherapy [23–25]. Apart from protein-encoding alterations, AS of 3′ untranslated regions (UTRs) is also upregulated in cancers and more prevalent in oncogenes [26]. The phenotypic outcome of aberrant AS may also mimic that of germline mutations without changing the genome. For instance, AS of DHX34 accounts for sporadic acute myeloid leukemia (AML) that phenocopies the loss-of-function mutation of DHX34 observed in familial AML cases [27].

Aberrant splicing factors are the major cause of global AS alteration in cancer. Expressional changes, mutation, and posttranslational modifications of splicing factors influence their catalytic activity or binding affinity with target RNAs. Specific splicing factors are most frequently mutated in certain cancer subtypes, and have been thoroughly reviewed elsewhere [4, 17]. While a vast majority of studies characterize how a single gene is modulated by upstream splicing factor alteration to promote cancer progression, the regulatory effects of splicing factors should be viewed as an entirety where splicing factors act on a set of genes to modulate certain aspects of tumor biology. Most splicing factors alter differently and elicit unique effects in different cancer types, but some have been recognized as universal oncoproteins or tumor suppressors. Serine/arginine-rich splicing factor 1 (SRSF1), as a representative, is upregulated in breast, colon, lung, and pancreatic cancer and uniformly promotes tumor progression [28–30]. Moreover, AS of splicing factors was also reported as a common feature across cancer types, adding to the complexity of the AS regulatory network [31]. DAP3, an oncogene overexpressed across cancer types, alters the AS pattern of multiple splicing factors, thereby coordinately modulating the splicing of downstream genes via their respective splicing factors to promote cancer progression [32].

Alterations of spliceosomal components were also seen in cancers. As a representative example, hotspot mutation of U1 spliceosomal small nuclear RNA (snRNA) (r.3a > *g*) is present in about 50% of Sonic hedgehog medulloblastomas [33]. This leads to a significant increase of alternative 5′ cryptic splicing events, including those concerning tumor suppressor genes, oncogenes, and hedgehog pathway effectors. In addition, inhibition of U1 snRNP function not only causes widespread premature transcription termination but also boosts cancer migration and invasion in vitro, suggesting a potent tumor suppressive role of U1 snRNP [34]. Attuning core spliceosome components is probably a potent measure to fix aberrant AS in cancers but must be applied with caution as it may also interfere with constitutive splicing in general.

## Circular RNA

### Biogenesis of CircRNA

CircRNA is generated through back-splicing (Fig. 1C). Canonical splicing joins an upstream 5′ splice donor site to a downstream 3′ splice acceptor site, resulting in the removal of the intron between adjacent exons. In cases of back-splicing, a downstream splice donor is joined to an upstream splice acceptor across one or more exons, forming a circular structure. Back-splicing utilizes the same spliceosome machinery as AS and competes with the linear splicing of cognate pre-mRNA [12, 35], but the efficiency of back-splicing is low compared with that of linear splicing owing to steric effects [36]. Biogenesis of circRNA is therefore mainly modulated by the kinetics of back-splicing.

Back-splicing is also regulated by cis-acting RNA sequences and trans-acting protein factors. Cis-acting elements play a prominent role in modulating the steric conformation of RNA molecules undergoing back-splicing. For instance, when flanked by the same intronic sequences, longer exons flavor circRNAs formation more than shorter ones for better feasibility of circular structure [12, 37]. Intronic complementary sequences (ICSs) in flanking introns play a prominent role in promoting circRNA biogenesis. ICSs at flanking introns base-pair to one another to bring distal splice sites into proximity, facilitating back-splicing [14, 37]. It has been reported that upon spliceosome inhibition, circRNAs with more distinct repeat sequences and more ICS motifs gain an expressional advantage [38]. Inverted repeated Alu (IRAlu) element is the most common ICS in humans and contributes the most to circRNA biogenesis [39]—trans-acting RBPs not only by binding to flanking intron but also by recognizing paired ICSs to regulate back-splicing. DExH-box helicase 9 (DHX9), a nuclear RNA helicase, unwinds IRAlu pairs to inhibit circRNA biogenesis [40].

Since most circRNAs rely on ICSs to form circular structures, the majority of circRNAs contain exons of their cognate mRNA or in some cases, introns retained between exons [41]. Rarely, lariat intermediate structures produced during pre-mRNA splicing give rise to circular intronic RNAs (ciRNAs) by the action of debranching enzymes [42].

The biogenesis of circRNA is better characterized concerning AS events. On the one hand, back-splicing itself can be viewed as an unusual type of AS event with unique circular products. On the other hand, all four canonical AS patterns, including the inclusion of cassette exons, intron retention, alternative 5′ splicing, and alternative 3′ splicing, have been identified in the back-splicing of multiple-exon circRNAs [8] (Fig. 1D). Alternative back-splicing events were identified for about 84% of circRNAs in a tissue-specific manner [43, 44], which is probably a result of tissue-specific expression of splicing regulatory factors. Further characterization of how the alternative back-splicing patterns are shifted in a particular disease context will provoke and expand current knowledge of circRNA-related pathogenesis.

### Molecular mechanism of CircRNA functions

CircRNAs are generally stable but weakly expressed compared with linear RNAs [45]. Owing to their circular structure, circRNAs are resistant to linear RNA decay machinery [36]. Though back-splicing takes place in the nucleus, exonic circRNAs are primarily localized in the cytoplasm by actions of nuclear export pathways [46, 47]. Intron-containing circRNAs, however, are mainly restrained in the nucleus to modulate transcription [41, 42].

In the cytoplasm, circRNAs interact with RNAs or proteins to exert their biological functions. Some circRNAs containing microRNA (miRNA) response elements (MREs) can act as competing endogenous RNAs (ceRNAs) to sponge miRNAs, sequestering miRNAs from binding to their target mRNAs [48]. While miRNA sponging is the most frequently presumed function of circRNAs, the majority of circRNAs contain fewer MREs than is expected [46], and the abundance of circRNAs should be taken into consideration when assessing their potential as ceRNAs [49, 50]. Apart from miRNAs, circRNAs can also interact with mRNAs to impact gene expression output [51]. CircRNAs engage with RBPs to modulate their behaviors. They can either act as scaffolds to recruit different proteins into proximity or function as protein sponges to block their function [52]. It is worth noting that a given circRNA may elicit composite regulatory roles in a certain context, and may elicit different physiological effects in a context-dependent manner.

Though circRNAs are classified as non-coding RNAs, emerging evidence has shown that circRNAs can serve as translational templates and the products of circRNA translation have important physiological and pathological correlations. Since circRNAs do not have a 5′ cap, internal ribosome entry sites (IRES) are required for circRNAs to undergo cap-independent translation [53]. Further study identified short IRES-like elements that also permit circRNA translation, expanding the range of possible circRNA translation events [54]. Furthermore, N^6^-methyladenosine (m^6^A) modification on circRNAs drives translation initiation by actions of m^6^A reader YTHDF3 and transcription factor eIF4G2 [55, 56]. The abundance of circRNA-produced peptides is limited by the low efficiency of cap-independent translation [57], but the distribution is characterized across multiple organs with implications of physiological importance [58]. Most protein products encoded by circRNA are unlikely to exhibit complicated biological functions as limited by their length and structural complexity but they may serve as potent agonists or antagonists once they contain corresponding conformational characteristics or binding sites.

### Regulation of CircRNA biogenesis in oncogenesis

Dysregulation of circRNA expression has been observed across cancer types. The global circRNA abundance is generally reduced in cancer cells owing to their rapid proliferation and low back-splicing efficiency [59, 60]. As back-splicing can happen in a co-transcription fashion, altered transcriptional status also influences circRNA biogenesis in cancer cells [61, 62]. Previous studies indicated that altered linear RNA synthesis modulates the relative amounts of linear RNA and circRNA. When the concentrations or activities of core spliceosome components are under restrictions, the full spliceosome packages across an exon owing to the limited transition from cross-exon interactions to cross-intron interactions, which induces the generation of circRNAs by back splicing [61]. Nevertheless, a plethora of studies have described the increased abundance of a given circRNA and its functional relevance in cancers. For example, ciRS-7, the first circRNA demonstrated as a miRNA sponge, is upregulated in multiple cancers and predicts poor prognosis [63–65].

Proteins encoded by circRNA may also participate in cancer progression. For example, circular E-cadherin encoded protein activates epidermal growth factor receptor (EGFR) independent of EGF to maintain glioma stem cell oncogenicity [66]. Thus, regulation of cancer-related circRNA may also proceed through translation, independent of its biogenesis. In HER2-positive breast cancer, circβ-TrCP encodes a truncated 343-amino acid peptide which binds to NRF2 and competitively blocks β-TrCP-mediated NRF2 degradation to promote trastuzumab resistance. In this case, the activity of circβ-TrCP is modulated by eIF3j transcriptionally without affecting circβ-TrCP biogenesis [67]. Similar to neo-junctions generated in AS events, back-splicing sites also impart immunogenicity to circRNA-produced proteins. A recent study illustrated that noncanonical translation of circRNAs can drive efficient antitumor immunity, implying the potential of utilizing tumor-specific circRNAs and their products as immunotherapeutic targets [68].

When investigating the biological function of circRNAs in cancers, the presence of stromal and immune components in the tumor microenvironment (TME) should not be ignored. As a representative example, the initial report on ciRS-7 suggested that ciRS-7 exerted oncogenic activity by sponging miR-7 inside cancer cells [63]. However, further study has found that ciRS-7 was not expressed in the cancer cells of several different adenocarcinomas and that the upregulation of ciRS-7 in tumors compared with adjacent normal tissues was attributed to its high expression in stromal cells in the TME [69]. The mechanism of ciRS-7-mediated oncogenesis should therefore be revised. Moreover, circRNAs packed into exosomes serve as an important intracellular crosstalk between different cellular components in the TME [70].

### Epigenetic and post-transcriptional modification concurrently regulates alternative splicing and CircRNA biogenesis in cancer

**Fig. 2:**
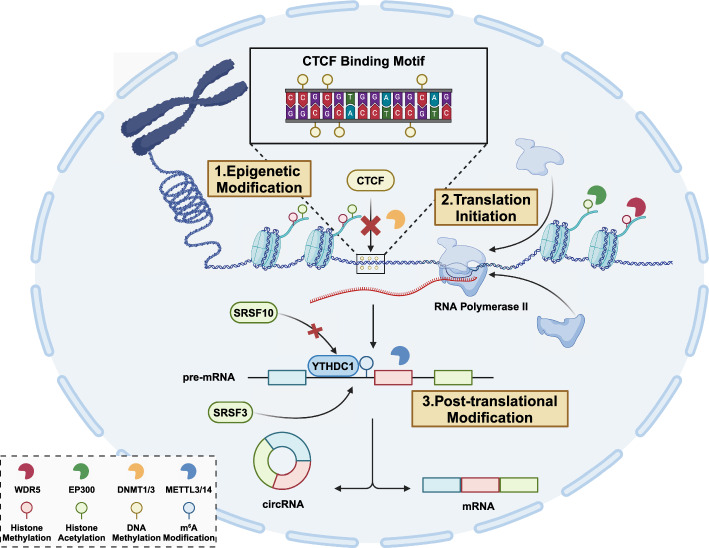
Epigenetic and posttranscriptional modifications concurrently modulating alternative splicing and circRNA biogenesis. Epigenetic modifications such as histone modification and DNA methylation regulates chromatin configuration to alter the accessibility of DNA, therefore influencing transcriptional activity and co-transcriptional splicing. Besides, histone or DNA methylation remarks create or shed binding sites of DNA binding proteins or the transcription machinery. Similarly, m^6^A modifications alter RNA structures or create RBP binding sites to regulate alternative splicing and circRNA biogenesis. CircRNA, circular RNA; CTCF, CCCTC-binding factor; METTL3/14, methyltransferase like 3/14; mRNA, messenger RNA; SRSF3/10, serine/arginine-rich splicing factor 3/10; YTHDF3, YTH N^6^-methyladenosine RNA binding protein F3

**Fig. 3:**
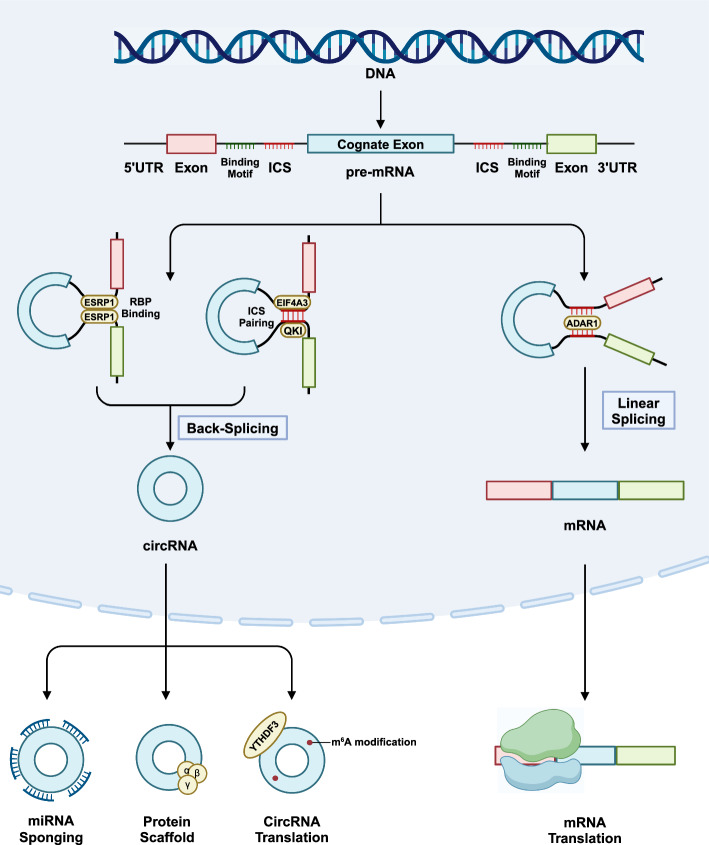
Regulation of circRNA biogenesis by RBPs. The majority of circRNAs originate form exons with ICSs or specific binding motifs in their flanking introns. Certain RBPs, such as ESRP1, recognizes its binding motifs to facilitate circularization of exons independent of other ICSs, such as Alu elements. Others, such as QKI and EIF4A3, on the other hand, attach to and stabilize formed ICS pairs. On the contrary, RNA helicase, such as ADAR1, unwinds ICS pairs to inhibit back-splicing and promotes linear splicing of pre-mRNAs. ADAR1, adenosine deaminases acting on RNA 1; circRNA, circular RNA; EIF4A3, Eukaryotic translation initiation factor 4A3; ICS, inverted complementary sequence; mRNA, messenger RNA; QKI, quaking

The interplay between AS events and circRNA in cancer has a rather straightforward interpretation, where AS as an upstream genetic event modulates the biogenesis of circRNAs to subsequentially influence downstream signal transduction in cancer cells, and circRNAs as signal molecules regulate the AS process of oncogenes to alter cellular behaviors. Apart from circRNAs and the AS machinery that compose the intricate regulatory network, epigenetic and posttranscriptional modifications controlling the transcriptome also concurrently regulate alternative splicing and circRNA biogenesis in cancers Fig. 2. Thus far, histone modification and m^6^A modification on pre-mRNAs have been revealed to intermediate the interaction between AS events and circRNAs Fig. 3.

### Histone modification

Histone modifications comprise a major part of epigenetic regulation. The fundamental unit of chromatin is the nucleosome, which consists of a central histone octamer wound by approximately 1.75 turns of DNA. Modifications on the core or the tail of histones affect the affinity of histone-DNA binding and hence impose direct or indirect effects on various DNA-templated processes [71–73].

Histone modifications emerge as a major regulator of AS events. Certain histone marks, such as H3-K36me3 and H3-K27me2, are identically enriched around exon splicing sites independent of nucleosome distribution and transcriptional activity [74]. Further study corroborated that distinctive histone modification signatures correlate with the AS outcome in a set of human genes, and histone marks possibly affect splicing outcomes by influencing the recruitment of splicing regulators via chromatin-binding protein [75]. Additionally, genome-wide screening of splicing-associated chromatin signatures suggested that histone modification signatures likely act in a combinatorial and position-dependent way and fine-tune the level of splicing variants rather than switching the splicing sites in a binominal fashion [76]. Identification of key histone-modifying enzymes or associated adaptor proteins is critical to resolving the intricate regulatory network between histone modifications and AS events. For instance, CHD8 suppression leads to a significant reduction in histone H3-K36me3 level, which correlates with altered alternative splicing patterns in autism spectrum disorders [77]. Intriguingly, a large subset of CHD8-related AS events are implicated in genes tagged with “regulation of RNA splicing,” and hnRNPs, especially hnRNPL, contribute to such alteration by interacting with CHD8. Therefore, the detailed mechanism by which histone modifications affect AS events requires further exploration and should be carefully distinguished from the global alteration of AS-related genes elicited by histone modifications.

Altered histone modification patterns and correlated AS events induce functional alterations in cancer cells. Dynamic changes in H3-K27ac and H3-K27me3 levels right at splicing sites are necessary and sufficient to induce AS events observed during epithelial-mesenchymal transition (EMT) with functional relevance recapitulating important aspects of EMT, such as cell motility and invasiveness [78]. Similarly, H3-K79me2 is involved in mediating exon-skipping events and interfering with DOT1L1, the sole H3-K79 methyltransferase, which impedes the proliferation of AML cells [79]. Small molecule inhibitors to selectively target the catalytic activity of histone-modifying enzymes have been applied to advanced tumor therapies in clinical trials [80, 81]. Furthermore, inhibiting H3-K9 methyltransferase SETDB1 leads to overexpression of newly-formed exon-TE splicing junctions in cancer cells to stimulate antitumor immunity, opening possibilities for tumor-targeting vaccination and novel immunotherapeutic targets [82].

Apart from linear splicing events, histone modifications are also implied in back-splicing events. Global histone modification pattern has been implied in circRNA biogenesis. Machine learning models based on histone modification patterns exhibited high accuracy in predicting circRNA expression in different cellular contexts [83]. Besides, circRNA abundance strongly correlates with response to histone acetylation inhibitors of cancer cells [84]. Alterations in specific histone modification sites may also induce alterations in corresponding circRNA biogenesis. In hepatocellular carcinoma, histone writer EP300 and WDR5 bind to the SOD2 promoter and trigger H3-K27ac and H3-K4me3 modification, respectively, which further activates circSOD2 expression [85]. EP300-mediated H3-K27ac also promotes the biogenesis of circCCAR1 in hepatocellular carcinoma cells [86]. Moreover, the expression of circRERE is facilitated by EP300, both of which are downregulated in colorectal cancer, but the corresponding histone modification signature has not been identified [87]. How aberrant histone modification pattern alters circRNA transcriptome and subsequentially induce phenotypic alterations in cancer requires further investigation.

### N^6^-methyladenosine modification

In eukaryotes, m^6^A modification is the most common RNA post-transcriptional modification, which involves the dynamic methylation of the sixth nitrogen (N) atom of RNA adenylate [88]. m^6^A modification of linear RNA is mainly carried out by the RNA methyltransferase complex, with methyltransferase-like-3 (METTL3) as the catalytic core and METTL14 as an RNA-binding platform [89].

Though m^6^A is not presupposed for most splicing events [90], some m^6^A readers are AS events and back-splicing that are functionally important. For instance, YTHDC1 promotes exon inclusion in targeted mRNAs through recruiting pre-mRNA splicing factor SRSF3 while blocking SRSF10 from accessing its binding regions [91]. Similarly, YTHDC1 and METTL3 are also reported to direct back-splicing events and regulate circRNA biogenesis [56]. Additionally, m^6^A also remodels RNA structure to alter its access to canonical splicing factors thereby influencing the choice of splicing sites [92].

Growing evidence revealed the essential role of m^6^A machinery in oncogenesis, progression, and therapeutic resistance [93–95]. By interacting or synergizing with other RBPs, m^6^a readers modulate circRNA biogenesis and thereby exert pleiotropic effects across various cancer types. In rhabdomyosarcoma, YTHDC1 and RNA helicase DDX5 serve as joint mediators of the back-splicing reaction, which promotes the generation of a shared subset of circRNAs thereby influencing cellular proliferation [96]. Likewise, the biogenesis of circTET2 is attuned by YTHDC1 along with splicing factor RBMX in chronic lymphocyte leukemia cells [97]. Subsequently, circTET2 interacts with HNRNPC to modulate downstream lipid metabolism and proliferation of chronic lymphocyte leukemia cells through the mTORC1 signaling pathway. By marking m^6^A sites in flanking reverse complementary sequences, METTL3 induces circ1662 in colorectal cancer, which enhances colorectal cancer invasion and migration through YAP1 and SMAD3 [98].

Hence, targeting m^6^A machinery therapy emerges as a potential approach for cancer treatment. Fusaric acid, a mycotoxin from *Fusarium* species, affects decreasing the expression of p53 by downregulating YTHDF1, YTHDC2, and YTHDF3 [99]. The bioactive small molecules extracted from natural sources exhibit unique chemical structures and diverse biological activities, which attributes natural products as potential sources for identifying novel therapeutic agents targeting m^6^A machinery.

### RNA binding proteins act in trans to modulate circRNA biogenesis

As highlighted earlier, RBPs are pivotal regulators of alternative splicing, including back-splicing events that govern circRNA biogenesis. In the context of circRNA formation, many RBPs act as trans-acting factors, influencing splice site selection and back-splicing efficiency. While most RBPs primarily function as transcription factors, recent evidence points to the involvement of RBPs with other roles—such as RNA base-pair editing proteins—in circRNA biogenesis. This diversity in RBP function underscores their multifaceted contributions to the regulation of RNA processing, linking alternative splicing and circRNA biogenesis in both normal cellular processes and cancer-related dysregulation.

### Adenosine deaminases acting on RNA

Adenosine deaminases acting on RNA (ADARs) protein family, including ADAR1 and ADAR2, participate in converting adenosines to inosines upon dsRNA binding [100, 101]. Differentially expressed ADARs and their aberrant A-to-I RNA editing are implicated in multiple cancer types [102–104]. Furthermore, IRAlu elements appear to be the most frequent and widespread targets of ADAR editing [105, 106], suggesting that ADARs hold great potential as potent circRNA regulators in cancer cells.

Previous studies deemed ADARs as putative circRNA inhibitors as they disrupt the formation of double-stranded RNA to repress Alu-mediated circRNA biogenesis and facilitate linear mRNA maturation [5, 107]. For instance, the androgen receptor could upregulate ADAR1 p110 to inhibit the production of circARSP91 in hepatocellular carcinoma and promote the growth of hepatocellular carcinoma [108]. ADAR2 was also suggested to inhibit circHif1a biogenesis and then allows miR-195a-3p to interfere with P-glycoprotein translation in breast cancer cells [109]. However, it has been recently revealed that the regulation of ADARs on circRNA biogenesis is bidirectional. Mechanistically, A-to-I editing can replace A: C mismatches with I(G)-C pairs or create I(G). U wobble pairs, which enables a more perfect dsRNA secondary structure and potentially facilitates circRNA production [110]. Beyond that, editing of ADARs may also create or modify auxiliary cis-acting elements for splicing factor binding, exemplified by the binding of PTBP1 to the flanking introns of circCHEK2 [110]. Despite that, the mechanisms determining the direction of circRNA regulation by ADARs remain unclear. Additionally, co-depletion of ADAR1 and DHX9 leads to a synergistic effect on circRNA biogenesis, implying extensive crosstalk between RBPs to be discovered [40].

### Quaking

Quaking (QKI), a KH domain-containing RNA binding protein, has been found to contribute to multiple malignant behaviors as a splicing factor [111]. Furthermore, QKI has been proposed as a master regulator of circRNA biogenesis. During the EMT process, QKI regulates not only pre-mRNA splicing but also the production of over one-third of abundant circRNAs [112]. The addition of QKI motifs alone is sufficient to induce de novo circRNA formation from linear transcripts, highlighting the potency of QKI as a regulator of back-splicing [112].

The activity of QKI is associated with cancer progression and therapeutic resistance. QKI can bind the introns of hsa_circ_0007919 pre-mRNA to increase the expression of hsa_circ_0007919, which enhances DNA damage response and promotes gemcitabine resistance in pancreatic ductal adenocarcinoma [113]. Apart from hsa_circ_0007919, QKI also promotes circARFGEF2 biogenesis by binding the specific motifs and neighboring reverse complement sequences in intron 3 and 6 of circARFGEF2 pre-mRNA [114]. CircARFGEF2 facilitates JAK2-STAT3 signaling by sponging miR-1205 to elicit lymph node metastasis in KRAS-mutant pancreatic ductal adenocarcinoma [114]. These findings indicate that QKI emerges as a potential therapeutic target to prevent pancreatic cancer progression. In esophageal cancer, QKI upregulates the expression of circBCAR3, which facilitates oncogenesis and metastasis of esophageal cancer [114]. In estrogen receptor-positive breast cancer, circSFMBT2 upregulated by QKI orchestrates ERα activation to drive tamoxifen resistance [115]. In response to radiation stimulation, TR4 in prostate cancer could transcriptionally boost QKI expression to increase circZEB1 levels, which then sponges the miR-141-3p to increase the expression of its host gene ZEB1, thereby strengthening resistance to radiotherapy [116]. In NSCLC, TGF-β treatment induces EMT and metastasis through QKI reduction, which decreased circ6834 expression [117].

However, in other contexts, QKI also exerts tumor-suppressive function and is recurrently repressed [118]. The expression of QKI-5 is reduced in hepatocellular carcinoma, which is responsible for the downregulation of circZKSCAN1 [119]. CircZKSCAN1 blocks the interactions between FMRP and CAR1 mRNA by binding FMRP competitively. Consequently, the Wnt/β-catenin signaling pathway is suppressed, which facilitates the hepatocellular carcinoma growth [119]. Downregulated QKI in non-small cell lung cancer contributes to the suppression of circNDUFB2. Functionally, circNDUFB2 inhibits non-small cell lung cancer progression through destabilizing IGF2BPs and activating antitumor immunity [120]. Moreover, in osteosarcoma, QKI inhibition leads to low circROCK1-E3/E4 expression, which suppresses osteosarcoma proliferation and migration by upregulating PTEN [121]. In addition, upregulation of QKI5 in cardiomyocytes significantly reversed doxorubicin-induced cardiotoxicity through upregulation of multiple circRNAs including circTTN, which is also implied in multiple cardiomyopathies [122–124]. Therefore, therapeutic attempts targeting QKI should further evaluate cardiac adverse response and rely on deeper delving into QKI function in different contexts.

### Eukaryotic translation initiation factor 4A3

Eukaryotic translation initiation factor 4A3 (EIF4A3) is a core component of the exon junction complex (EJC). As an entirety, the EJC plays an essential role in suppressing aberrant AS events to maintain transcriptome integrity and facilitates various genetic processes including mRNA transport and NMD [125–128]. Beyond that, EIF4A3 acts as a pre-mRNA splicing factor to modulate AS with robust implications for oncogenesis [129, 130].

Numerous reports have illustrated that EIF4A3 regulates back-splicing to regulate circRNA biogenesis (Table 1). Generally, EIF4A3 modulates circRNAs to impose pleiotropic effects on cancer cells with functional significance. Part of them mediates major oncogenic pathways to affect cancer progression. In breast cancer, EIF4A3 promotes circIKBKB biogenesis, which activates NF-κB signaling to induce osteoclast differentiation and bone metastasis [131]. Blocking EIF4A3 pharmaceutically inhibits the circIKBKB biogenesis and bone-metastasis of breast cancer, which provides a novel therapeutic target for breast cancer treatment [131]. EIF4A3 also promotes the biogenesis of circSEPT9 which facilitates the oncogenesis and progression of triple-negative breast cancer by activating activates STAT3 signaling [132]. EIF4A3 promotes the biogenesis and cytoplasmic export of circARHGAP29, which increases the stability of LDHA and c-Myc mRNA to enhance the glycolytic metabolism and confer docetaxel resistance in prostate cancer [133]. Additionally, increased circASAP1 by EIF4A3 boosts the expression of NRAS via sponging miR-502-5p to promote proliferation and temozolomide resistance of glioblastoma [134]. In osteosarcoma, EIF4A3 binds to one downstream flanking sequence of PRKAR1B pre-mRNA and promotes back-splicing of circPRKAR1B. circPRKAR1B subsequentially promotes osteosarcoma progression by modulating the expression of FZD4 and hyperactivating Wnt signaling [135]. The binding of EIF4A3 to the flanking sequence may also prevent back-splicing of certain tumor-suppressive circRNAs. For instance, EIF4EA3 suppresses circ0009092 expression in colorectal cancer cells. Circ0009092 elevates NLK expression via sponging miR-665, which inactivates the Wnt/β-catenin signaling pathway and regulates STAT3 signaling to inhibit macrophage in the TME [136]. EIF4A3 also suppresses the cyclization of circPTEN1 by directly binding to its flanking region in colorectal cancer. Mechanistically, circPTEN1 is capable of disrupting Smad signaling and consequently suppresses the EMT process [137]. Moreover, circ0087429 can reverse EMT and inhibit the progression of cervical cancer by upregulating OGN expression, which is suppressed by EIF4A3 [138].

**Table 1:** CircRNAs modulated by EIF4A3

Cancer	circRNA	circRNA function	Tumor behavior	Reference
CircRNAs upregulated by EIF4A3				
Breast cancer	circPRKCI	miRNA sponge (miR-545-3p)	Pro-tumor, proliferation, apoptosis, metastasis	[221]
	circ_0042881	miRNA sponge (miR-217)	Pro-tumor, proliferation, metastasis,	[222]
	circSEPT9	miRNA sponge (miR-637)	Pro-tumor, proliferation, migration, invasion	[132]
	circSERPINE2	miRNA sponge (miR-513a-5p)	Pro-tumor, tumor microenvironment	[142]
	circZFAND6	miRNA sponge (miR-647)	Pro-tumor, proliferation, metastasis	[223]
	circBRWD3	miRNA sponge (miR-142-3p and miR-142-5p)	Pro-tumor, proliferation, migration, invasion	[224]
	circWAC	miRNA sponge (miR-599)	Pro-tumor, proliferation, apoptosis, migration, invasion, glycolysis	[225]
	hsa_circ_0088088	miRNA sponge (miR-135-5p)	Pro-tumor, proliferation, metastasis	[226]
	circIKBKB	Protein binding	Pro-tumor, metastasis	[131]
Bladder cancer	circSTX6	miRNA sponge (miR-515-3p), protein binding	Pro-tumor, metastasis, therapy sensitivity (cisplatin)	[227]
	circ0008399	Protein binding	Pro-tumor, apoptosis, therapy sensitivity (cisplatin)	[145]
Cholangiocarcinoma	circZNF609	miRNA sponge (miR-432-5p)	Pro-tumor, proliferation, migration, invasion	[228]
Colorectal cancer	circ_0084615	miRNA sponge (miR-599)	Pro-tumor, proliferation, migration, invasion, angiogenesis	[229]
Gastric cancer	circGLIS3	miRNA sponge (miR-1343-3p), protein binding	Pro-tumor, proliferation, metastasis	[230]
	circABCA5	Protein binding	Pro-tumor, proliferation, migration, invasion	[231]
	hsa_circ_001988	miRNA sponge (miR-197-3p)	Antitumor, proliferation, metastasis	[232]
Glioma	circGRIK2	miRNA sponge (miR-1303)	Pro-tumor, proliferation, migration, invasion	[233]
	circCCNB1	miRNA sponge (miR-516b-5p), protein binding	Pro-tumor, proliferation	[234]
Glioblastoma	circASAP1	miRNA sponge (miR-502-5p), protein binding	Pro-tumor, proliferation, therapy sensitivity (temozolomide)	[134]
	circMMP9	miRNA sponge (miR-124)	Pro-tumor, proliferation, migration, invasion	[235]
Hepatocellular carcinoma	circCCAR1	miRNA sponge (miR-127-5p)	Pro-tumor, proliferation, metastasis, T cell exhaustion, therapy sensitivity (anti-PD1)	[86]
	circCDYL	Protein binding	Pro-tumor, metastasis, EMT	[236]
	circTOLLIP	miRNA sponge (miR-516a-5p)	Pro-tumor, proliferation, metastasis, EMT	[237]
	circRHBDD1	Protein binding	Pro-tumor, proliferation, glycolysis, therapy sensitivity (anti-PD1)	[141]
Lung cancer	circRABL2B	Protein binding	Antitumor, proliferation, metastasis, stemness, therapy sensitivity (erlotinib)	[238]
	circSCAP	miRNA sponge (miR-7)	Pro-tumor, proliferation, metastasis	
Nasopharyngeal carcinoma	circFIP1L1	miRNA sponge (miR-1253)	Pro-tumor, proliferation, apoptosis, therapy sensitivity (radiotherapy)	[239]
Osteosarcoma	circPRKAR1B	miRNA sponge (miR-361-3p)	Antitumor, proliferation, metastasis, EMT, therapy sensitivity (cisplatin)	[135]
Pancreatic cancer	circRNF13	miRNA sponge (miR-654-3p)	Pro-tumor, proliferation, angiogenesis, invasion, glycolysis	[240]
Prostate cancer	circTFDP2	Protein binding	Pro-tumor, proliferation, metastasis	[241]
	circARHGAP29	Protein binding	Pro-tumor, proliferation, angiogenesis, invasion, glycolysis	[133]
		miRNA sponge (miR-133a-3p and miR-133b)	Antitumor, therapy sensitivity (enzalutamide)	[242]
CircRNAs downregulated by EIF4A3				
Cervical cancer	circ_0087429	miRNA sponge (miR-5003-3p)	Antitumor, proliferation, metastasis, angiogenesis, EMT	[138]
Colorectal cancer	circβ-catenin	miRNA sponge (miR-197-3p)	Pro-tumor, proliferation, metastasis	[243]
	circ_0009092	miRNA sponge (miR-665)	Pro-tumor, proliferation, invasion, TAM recruitment	[136]
	circPTEN1	Protein binding	Antitumor, metastasis, EMT	[137]
Gastric cancer	circ_100290	miRNA sponge (miR-29b-3p)	Pro-tumor, proliferation, migration, invasion, EMT	[244]
Laryngeal squamous cell carcinoma	hsa_circ_0001445	miRNA sponge (hsa-miR-432-5p)	Pro-tumor, proliferation, migration, invasion	[245]
Lung cancer	circ_0067716	miRNA sponge (miR-324-5p), protein binding	Anti-tumor, proliferation, apoptosis	[246]

While the majority of studies depicted the oncogenic potentials of EIF4A3, it has also been reported that EIF4A3 regulates circRNAs to exert tumor-suppressive effects. In hepatocellular carcinoma, circGLIS2 inhibits EMT and cellular proliferation, migration, and invasion [139]. It is found in this study that upregulation of EIF4A3 elevated circGLIS2 expression, but as the results also demonstrated that EIF4A3 promotes the transcription of the cognate GLIS2 mRNA expression, whether EIF4A3 directly modulates back-splicing to promote circGLIS2 biogenesis remains elusive and requires further validation. In NSCLC, EIF4A3 was found to decrease the expression of circ_0007386, an oncogenic circRNA, by binding to its cognate CRIM1 pre-mRNA [140]. Intriguingly, under hypoxic conditions, increased YAP1 binds to EIF4A3, which leads to the displacement of EIF4A4 from CRIM1 pre-mRNA and facilitates the biogenesis of circ_0007386 [140].

Furthermore, some EIF4A3-modulated circRNAs exhibit immunomodulatory properties and shape the TME. EIF4A3 and E1A binding protein p300 (EP300) synergistically promote the biogenesis of circCCAR1 in hepatocellular carcinoma. Exosomal circCCAR1 is taken in by CD8^+^ T cells in the TME and disables CD8^+^ T cells by stabilizing the PD-1 protein [86]. Though a clear mechanism remains elusive, EIF4A3-induced circRHBDD1 is also upregulated in hepatocellular carcinoma patients and predicts poor response to anti-PD-1 treatment [141]. In addition, EIF4A3 promotes the formation of circSERPINE2 in breast cancer, which is shuttled to tumor-associated macrophages through exosomes and enhances the secretion of Interleukin-6, leading to increased proliferation and invasion of breast cancer cells [142].

The interplay between the m^6^A modification machinery adds to the complexity of EIF4A3-based regulatory pathways. EIF4A3 deposition and concurrent EJC assembly are involved in controlling m^6^A modification via steric blockade of METTL3, and depletion of EIF4A3, leads to increased m^6^A modification at sites close to exon-exon junctions within mature mRNA [143]. Moreover, circRNAs induced by EIF4A3 impose bidirectional effects on m^6^A modification. EIF4A3-induced circ0001187 promotes proteasomal degradation of METTL3, suppressing AML progression [144]. However, circ0008399, elevated by EIF4A3 in bladder cancer, binds WTAP to promote the formation of the WTAP-METTL3-METTL14 m^6^A methyltransferase complex, which stabilizes TNFAIP3 mRNA in an m^6^A-dependent manner to promote cisplatin resistance [145]. Similarly, circRHBDD1 recruits the m^6^A reader YTHDF1 to PIK3R1 mRNA and accelerates the translation of PIK3R1 in an m^6^A-dependent manner [141]. Therefore, instead of measuring how EIF4A3 affects the global m^6^A modification level, characterizing the m^6^A modification status of genes with functional relevance may better reflect its role in cancers.

Notably, EIF4A3 also serves as a transcription factor and a subset of endogenous circRNAs are subject to EIF4A3-mediated transcription, some of which even express a full-length intact protein, such as β-catenin [146]. Therefore, EIF4A3 and other EJC components possibly serve as a potent circRNA regulator that remains to be further characterized.

### Epithelial splicing regulatory protein 1

Epithelial splicing regulatory protein 1 (ESRP1) is an epithelial cell-specific RBP that contributes to the regulation of AS and translation AS events elicited by ESRP1 are actively engaged in organogenesis by modulating the transition between mesenchymal and epithelial cellular states [147]. As is expected, ESRP1 also orchestrates AS events in multiple cancer types and is suggested as a tumor-suppressive splicing factor that represses EMT to restrain malignancy [148, 149]. Current evidence has expanded its role in circRNA biogenesis. ESRP1 promotes the formation of circDOCK1(2–27) by binding and splicing a GGU-containing repeat region in intron 1 during EMT of breast cancer cells. CircDOCK1(2–27) represses cell motility and migration by diverting transcripts from DOCK1 mRNA to circRNA [150]. Moreover, ESRP1 is involved in the biogenesis of circ-TNPO3, which significantly suppresses the proliferation and migration of clear cell renal cell carcinoma cells [151]. In hepatocellular carcinoma, ESRP1 was identified to bind to the pre-mRNA of PTPN12, thereby fostering the generation of circPTPN12 to suppress tumor progression [152]. Moreover, ESPR1 also promotes the biogenesis of circBIRC6 to control the molecular circuitry in human embryonic stem cells, which regulates cell pluripotency and early lineage differentiation [153]. However, it has been reported that ESPR1 and pre-mRNA methylation of NOL10 jointly inhibit the biogenesis of circNOL10 to promote the development of lung cancer [154]. Similarly, ESRP1 has been suggested to aggravate prostate cancer progression and independently predicts poor prognosis of prostate cancer patients [155–157]. The contradictive effects of ESPR1 in different cancer types possibly stem from the distinctive expressional context of the original tissue, which should be taken into consideration when designing ESPR1-based therapies.

### Heterogeneous nuclear ribonucleoprotein particle proteins

As mentioned above, hnRNP is a class of crucial splicing factors, which is implicated in the diverse RNA transcript and protein biogenesis across the progression of multiple cancer types. Among hnRNP family members, HNRNPL is most frequently implied in circRNA alteration in cancers. Genome-wide CRISPR screens have identified its essential role in the growth of prostate cancer by regulating alternative RNA splicing and circular RNA formation [158]. In bladder cancer, HNRNPL promotes the circFAM13B biogenesis through pre-mRNA back-splicing, which competitively binds to the KH3-4 domains of IGF2BP1 and subsequently decreases the binding of IGF2BP1 to the PKM2 3′UTR; thereby weakening PKM2 mRNA stability and reversing glycolysis-induced acidic tumor microenvironment [159]. In hepatocellular carcinoma, HNRNPL promotes circARHGAP35 biogenesis to facilitate the formation of an oncoprotein [160]. The interactions of circARHGAP35 oncoprotein and TFII-I protein in the nucleus promote cell progression of hepatocellular carcinoma [160]. These findings indicate the diverse roles of HNRNPL and its isoforms in RNA processing, alternative splicing, and circular RNA biogenesis in cancer pathogenesis. Disruptions of the splicing machinery of HNRNPL emerge as a potential approach to inhibiting cancer progression.

Apart from HNRNPL, other hnRNP members are also proven as circRNA regulators in cancer. HNRNPM preferentially binds to GU-rich elements in long-flanking proximal introns to prevent aberrant exon inclusion and back-splicing events. Loss of HNRNPM results in generally increased circRNA formation and inhibits prostate cancer cell growth [161].

### Fused in sarcoma

Fused in sarcoma (FUS) is a classical regulator involved in DNA replication, translation, and repair [162–164], which plays a critical role in cell proliferation and tumor progression [165]. Recently, several studies investigated the effect of FUS on the circRNA biogenesis in multiple cancer types. In triple-negative breast cancer, FUS facilitates the cyclization of circTBC1D14 by binding the downstream flanking sequence of circTBC1D14, which promotes the progression and aggressiveness of triple-negative breast cancer in vivo [166]. Moreover, hypoxic conditions can increase the level of FUS-circTBC1D14-associated stress granules in the cytoplasm. The circTBC1D14 is mostly increased in triple‐negative cell lines and associated with poor prognosis of breast cancer [166]. In renal cell carcinoma, FUS contributes to converting fibroblasts to cancer-associated fibroblasts by the biogenesis of circEHD2 [167]. CircEHD2 and YAP bind to the SOX9 promoter and activate the expression SOX9 with the help of YWHAH, and consequently facilitate the proliferation and migration of renal cell carcinoma [167]. In colorectal cancer, FUS binds to specific motifs and Alu elements in the introns flanking the exons of circRHOBTB3 along with ADARB2 to promote the biogenesis of circRHOBTB3 [168]. CircRHOBTB3 is a novel metastasis-related factor in colorectal cancer by binding to the functional RNA-binding protein human antigen R (HuR). The interaction between circRHOBTB3 and HuR promotes HuR degradation by accelerating the β-Trcp1-mediated ubiquitination of HuR, which represses PTBP1-mediated metastasis in renal cell carcinoma [168].

### NOVA2

NOVA2 is a neuron-enriched splicing factor that plays a crucial role in the development of the central nervous system and synaptic plasticity [169, 170]. Mechanistically, NOVA2 recognizes clusters of tetranucleotide YCAY motifs and recruits CPSF and SYMPK to facilitate the early binding of spliceosome components [171]. Apart from its regulatory role in AS events, NOVA2 globally promotes circRNA biogenesis in the developing brain [172]. Linear splicing and back-splicing are likely modulated by NOVA2 in parallel, as NOVA2-regulated circRNAs and AS events show little overlap [172]. In glioma, circU2AF1 upregulates NOVA2 by sponging hsa-miR-7-5p to aggravate malignancy and predict poor prognosis of patients [173]. Beyond its role in the central nervous system, NOVA2 also participates in the regulation of endothelial function [174, 175]. Current studies have corroborated the oncogenic potential of NOVA2 by stimulating angiogenesis in different malignancies [176–178]. Remarkably, multiple studies have reported a specific circRNA regulates lung cancer progression through NOVA2, but the exact AS alteration or other possible molecular alterations related to NOVA2 have not been thoroughly examined in the context of lung cancer [179–181].

Notably, each cicrRNA may have distinct cancer-related functions in specific cancer types. We should note that the contribution of splicing factor-regulated circRNA biogenesis to cancer is unlikely to be attributed to a single signaling pathway. Further studies should concentrate on the crosstalk between different splicing factors and circRNAs in specific cancer types.

### CircRNAs regulate alternative splicing of cancer-related genes

#### CircRNAs modulate splicing factor functions

**Fig. 4:**
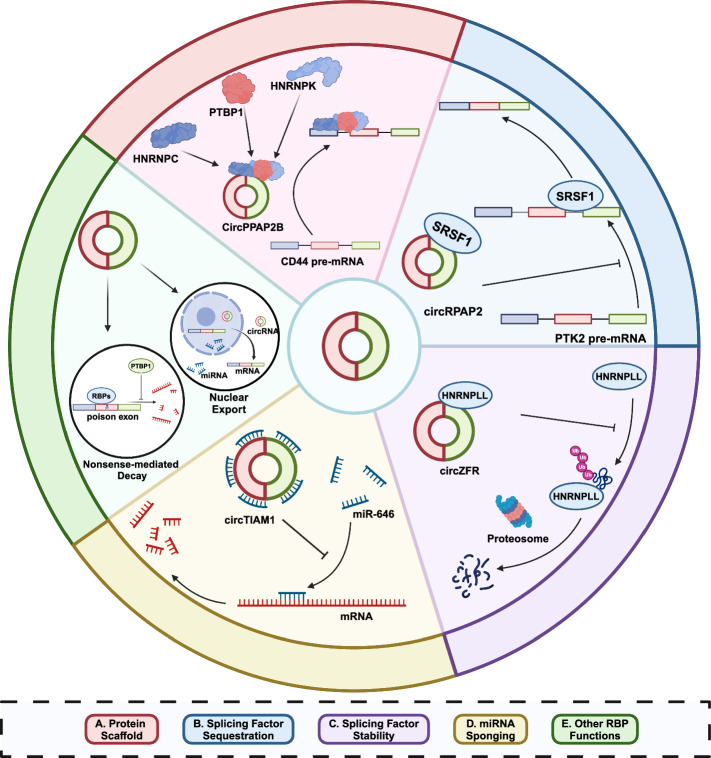
CircRNAs modulate splicing factor functions. CircRNAs exert their molecular functions to regulate splicing factor functions and alternative splicing. The binding between circRNA and splicing factors can either facilitate the assembly of protein complexes (**A**) or sequester their function by competing with target RNAs (**B**). In addition, such binding may also influence the stability of splicing factors by altering their accessibility to protein degradation machineries (**C**). As miRNA sponges (**D**), circRNAs also regulate the expression of splicing factors. As some splicing factors also participates in other RBP functions such as nonsense-mediated decay and nuclear export (**E**), the interaction between splicing factors and circRNAs carries even greater significance. CircRNA, circular RNA; HNRNP, heterogeneous nuclear ribonucleoprotein particle; mRNA, miRNA, microRNA; messenger RNA; RBP, RNA binding protein; SRSF1, serine/arginine-rich splicing factor 1, Ub, Ubiquitin

CircRNAs modulate AS events mainly through their interactions with splicing factors (Fig. 4). The binding between circRNA and splicing factors can directly influence the regulatory function of the corresponding splicing factor. CircRNAs may serve as decoys targeting splicing factors to block their effects on AS. In gastric cancer, circURI1 binds and sequesters hnRNPM to modulate AS of genes involved in the process of cell migration, thus suppressing GC metastasis, demonstrated by the fact that circURI1 influenced exon inclusion or exclusion in contrast to hnRNPM [182]. CircRAPGEF5 confers ferroptosis resistance to endometrial cancer cells through the interaction with RBFOX2. CircRAPGEF5 can bind to the Fox-1 C-terminal domain of RBFOX2 and induce specific exon exclusion of TFRC by obstructing the binding of RBFOX2 to pre-mRNA [183]. The binding of circRNA could compete with the target pre-mRNA of splicing factors. For instance, circRPAP2 can bind to the oncogenic splicing factor SRSF1 to compete with the binding between SRSF1 and PTK2 pre-mRNA, thereby attenuating SRSF1-mediated alternate splicing of PTK2 and reduce its expression in breast cancer [184]. Alterations elicited by circRNA binding could also gain functional advantage for certain splicing factors owing to possibly conformational changes. In gallbladder cancer, circFOXP1 binds with PTBP1 and enhances the capacity of PTBP1 to bind to PKLR mRNA [185]. PTBP1 can bind to the 3′UTR region and coding region of PKLR mRNA to protect PKLR mRNA from NMD [186].

CircRNAs as protein scaffolds can recruit RBPs and thereby assemble functional complexes or bring their targets into proximity. In clear renal cell carcinoma, circPPAP2B is significantly upregulated compared with adjacent normal tissue and promotes cancer metastasis [187]. CircPPAP2B facilitates HNRNPC nuclear translocation in an m^6^A-dependent manner and recruits PTBP1 and HNPNPK as a complex to regulate pre-mRNA splicing. Consequent AS events include the skipped exon 8 of CD44, a critical cell-surface glycoprotein involved in cell adhesion and metastasis. In gastric cancer cells, hsa_circ_0000437 binds SRSF3 and inhibits PDCD4 to promote cell proliferation, invasion, migration, and apoptosis [188]. It is proposed that hsa_circ_0000437 recruits SRSF3 and possibly forms a triplet complex PDCD4 mRNA and inhibits the translation of the tumor suppressor gene PDCD4. However, such a mechanism requires further validation as the original study provided scarce information about the putative complex. Moreover, according to a previous report, SRSF3 not only regulates the alternative splicing pattern of PDCD4 pre-mRNA but also directly modulates its translational efficiency in the cytoplasm [189]. This highlights the fact that circRNA functions should be investigated with caution and resolve possible protein-RNA and protein–protein interactions.

The stability of splicing factors can be regulated by circRNAs by exposing or shielding target spites of protein degradation machinery. In lung cancer cells, circZFR protects HNRNPLL from degradation by ubiquitination to regulate AS of target genes, such as myosin IB, and subsequently activates the AKT-mTOR pathway to facilitate oxidative phosphorylation [190]. Conversely, circSCAP directly binds to SF3A3 to facilitate its ubiquitination, which enhances the expression of MDM4 exon 6 skipping transcripts to finally activate downstream p53 signaling in non-small cell lung cancer [191].

The interaction between circRNAs and splicing factors does not necessarily affect splicing events corresponding to a certain phenotypic alteration as manifested by the versatility of RBPs. In intrahepatic cholangiocarcinoma, circMBOAT2 is upregulated and promotes cancer progression by stabilizing PTBP1 to facilitate FASN mRNA cytoplasmic export, which alters lipid metabolic profile and regulates redox homeostasis [192]. Data from the study showed that the splicing form of FASN was not changed after knocking down PTBP1 but an examination of AS events at the whole-transcriptome level was absent. Further studies characterizing how circRNAs affect global splicing events will provide more details with higher fidelity on how circRNAs participate in the regulatory network of splicing factors.

CircRNAs with microRNA sponging function could regulate the expression level of splicing factors to further modulate AS events. In non-small cell lung cancer, circRNA_100146 is identified to bind different subtypes of splicing factor SF3 family and may affect SF3B3 expression through sponging miR-361-3p and miR-615-5p [193]. In papillary thyroid cancer, circTIAM1 acts as a sponge of miR-646 to promote oncogenesis by elevating HNRNPA1 expression [194]. It is worth noting that circRNAs may cause phenotypic alteration through parallel protein interacting and microRNA sponging mechanisms. For instance, circPPAP2B not only serves as a protein scaffold as mentioned above, but also sponges miR-182-5p to increase CYP1B1 expression in the same context [187].

### Feed-back regulation loops of CircRNAs

As discussed above, the biogenesis of circRNAs is also modulated by multiple splicing factors. Therefore, the functional relevance between splicing factors and circRNAs is bidirectional and possibly manifests in a feedback loop regulation. Most commonly, oncogenic circRNAs are involved in positive feedback loops that are self-augmenting and progressively promote cancer progression. On the contrary, negative feedback loops of circRNAs are self-limiting and play a role in maintaining the homeostasis of its components. Perturbations from oncogenic factors may predispose, such loops toward a harmful level. In both cases, the loop itself is irrelevant to particular phenotypes but malfunction of its components alters cellular behaviors and thereby exerts oncogenic effects.

#### ADAR

In pancreatic ductal adenocarcinoma, circNEIL3 was shown to boost the expression of ADAR1 by sponging miR-432-5p to antagonize miR-432-5p-induced ADAR1 degradation [195]. In return, ADAR1 was shown to decrease circNEIL3 abundance, forming a negative feedback loop that holds the balance between circNEIL3 and ADAR1. Upregulation of ADAR1 subsequentially induces RNA editing of GLI1, ultimately influencing the cell cycle and promoting EMT in pancreatic ductal adenocarcinoma cells. However, further validations from clinical data in this study indicated that circNEIL3 and ADAR1 expression were negatively correlated with miR-432-5p expression levels, while circNEIL3 was positively correlated with ADAR1. Though interpretation of such controversy requires further experimental exploration, we could conclude from this case that dynamic molecular mechanisms may or may not be reflected in a cross-sectional clinical observation that represents an already-built disease state at a single time point.

#### hnRNP family

In gastric cancer, circFAM73A promotes the expression of HMGA2 by sponging miR-490-3p [196]. Upregulated HMGA2 not only boosts cancer stemness but also enhances HNRNPL activity, which in turn promotes circFAM73A biogenesis to build up a positive feedback loop. Intriguingly, HMGA2 also facilitates the transcription of cognate FAM73A mRNA by increasing the activity of transcription factor E2F1. As a consequence, the increase of circFAM73A does not accompany significant alteration in its cognate mRNA transcripts. A similar regulatory process was also identified for circTDRD3. In colorectal cancer, circTDRD3 elevates HIF1α expression by sponging miR-1231 [197]. HIF1α synergistically induces the transcription of TDRD3 pre-mRNA and PTBP1 to accelerate the formation of circTDRD3. As the oncogenic effector, HIF1α consequently facilitates colorectal cancer proliferation and metastasis. In both cases, the loops are joined by transcription factors that elevate the expression of both splicing factors and their target gene. Considering the possible effects from the expression of cognate mRNAs, targeting splicing factors to suppress cancer progression seems less favorable than targeting other nodes on the chain.

#### FUS

As mentioned above, FUS is involved in circRNA biogenesis by binding flanking introns to facilitate circularization. In breast cancer, FUS is implied in multiple positive feedback loops concerning circRNA biogenesis, highlighting its potential as a therapeutic target. CircROBO1 could upregulate KLF5 by sponging miR-217-5p, allowing KLF5 to activate the transcription of FUS, which in return promotes the back splicing of circROBO1 [198]. Besides, overexpressed circEZH2 was reported to promote tumorigenesis and liver metastasis of breast cancer in vivo. Mechanistically, circEZH2 also adsorbs miR-217-5p to upregulate KLF5 thus leading to FUS transcription [199]. Another report concerning triple-negative breast cancer indicated that FUS could regulate the biogenesis of circHIF1A and that FUS was transcriptionally regulated by NFIB [200]. CircHIF1A promotes the expression and translocation of NFIB through posttranscriptional and posttranslational modifications. While present studies have well elucidated the pluripotency of FUS, a thorough assessment of how FUS influences circRNA transcriptome in breast cancer will consolidate the feasibility of exploiting FUS as a therapeutic target.

#### ESRP1

ESRP1 is actively involved in cancer progression by mediating the EMT process and ESRP1-related regulatory loops were well depicted. In oral squamous cell carcinoma, circUHRF1 acts as the sponge of miR-526b-5p, thereby upregulating transcription factor c-Myc. c-Myc could accelerate the transcription of ESRP1 and other oncogenes. Moreover, ESRP1 promoted the circularization and biogenesis of circUHRF1 by targeting the flanking introns, forming the circUHRF1/miR-526b-5p/c-Myc/ESRP1 positive feedback loop [201]. Moreover, circANKS1B biogenesis in breast cancer is promoted by ESRP1. circANKS1B serves as a miRNA sponge to increase the expression of transcription factor USF1, which elevates the expressional level of USF1 and EMT-related genes [202].

Intriguingly, the cognate circRNA of ESRP1 inhibits EMT clear cell renal cell carcinoma by attuning c-Myc activity. CTCF, a downstream target of miR-3942, specifically promotes circESRP1 transcription and is regulated by the circESRP1/miR-3942 pathway to form a positive feedback loop [203]. Though the study reported no significant correlation between CTCF and ESRP1 mRNA, given that ESRP1 itself is involved in the regulation of circRNA biogenesis across multiple cancer types including clear cell renal cell carcinoma [151], it is conceivable that circESRP1 and the splicing factor ESRP1 is involved in complex regulatory network that remains unresolved.

#### Zinc finger E-box binding homeobox 1

Zinc finger E-box binding homeobox 1 (ZEB1) is a major EMT regulating transcription factor that acts upstream of multiple splicing factors, including ESRP1 and EIF4A3, to repress their expression [204, 205]. Hence, alteration of ZEB1 may reshape circRNA biogenesis indirectly through its transcriptional activity. Beyond that, current studies have corroborated that ZEB1 directly regulates circRNA biogenesis to affect carcinogenesis. CircNIPBL activates the Wnt/β-catenin signaling pathway via directly sponged miR-16-2-3p, leading to the upregulation of ZEB1, which triggers the EMT of bladder cancer. ZEB1 interacted with the flanking introns on NIPBL pre-mRNA to trigger circNIPBL biogenesis, thus forming a positive feedback loop [206]. Besides, ZEB1 also downregulates circDOCK5 through both directly binding to its pre-mRNA and suppressing EIF4A3 expression in esophageal squamous cell carcinoma. CircDOCK5 increased the stability of miR-627-3p by functioning as a “reservoir” for miR-627-3p to partially reverse the ZEB1-enhanced migration and invasion. MiR-627-3p inhibited the expression of TGFB2 and the secretion of TGF-β, which further resulted in downregulation of ZEB1 and suppression of TGF-β-induced EMT [205].

## Conclusions

**Fig. 5:**
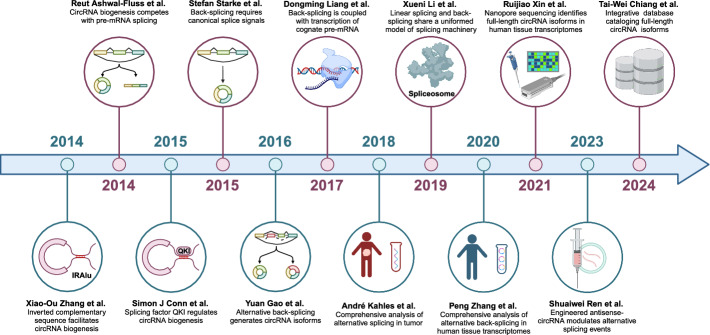
Remarks of studies demonstrating the crosstalk between circRNA and alternative splicing in cancer. During the past decade, blooming studies have well established the crosstalk between circRNA and alternative splicing in cancer. Ever since the initial insight into the biogenesis of circRNA by Zhang et al. and Ashwal-Fluss et al. in 2014, dedicated studies have indicated the tight relationship between AS and circRNAs. As the molecular mechanisms of circRNA biogenesis and alternative splicing are resolved, it has been widely recognized that circRNA and alternative splicing are coupled through key steps in gene expression. Nowadays, nanopore sequencing technology has laid foundation for genomic-scale profiling of full-length mRNA and circRNA splicing variants, which provide valuable resources for understanding cancer biology

Ever since the initial insight into the biogenesis of circRNA by Zhang et al. and Ashwal-Fluss et al., blooming and dedicated studies have revealed the tight relationship between AS and circRNAs and their roles in cancer biology (Fig. 5) [14, 35, 207]. The interplay between circRNAs and AS underscores a complex regulatory mechanism, where AS coordinates circRNA biogenesis and splicing of their linear cognate transcripts, and circRNAs interact with components of the splicing machinery to modulate AS events. Moreover, modifications on DNA, pre-mRNA, or histones simultaneously affect AS and circRNA biogenesis by establishing binding motifs or steric effects. Such intricate crosstalk between circRNAs and AS represents a critical facet of gene regulation in cancer biology. Malfunctioning AS machinery results in the anomalous transcriptome, including circRNA transcriptome, that underlies tumor progression, relapse, and therapy resistance. Currently, dedicated works illustrating how circRNAs and AS interact at a single gene level have been emerging, but inclusive and systematic research to draw the whole picture is still lacking. As we emphasized above, feedback regulations between AS and circRNAs reflect the potential homeostasis of circRNA biogenesis that is recurrently disrupted in cancers. Additionally, the versatility of circRNAs and RBPs implies that a multilayer and interlaced regulatory network will surely fit better than a model depicting isolated regulatory axes. Therefore, utilizing a multiomic approach to investigate the relationship between circRNA will provide better insight into cancer biology.

Current advances in nanopore-based long-read sequencing have enabled reliably characterization of full-length mRNA and circRNA isoforms [208, 209], laying the foundation for databases with comprehensive RNA variant catalogs [210]. Despite that, the low abundance of circRNA and AS products still obscures genomic scale studies. For instance, though it has been well illustrated that alternative back-splicing generates circRNA isoforms, scarcely have studies identified or distinguished circRNA variants with functional relevance. Similarly, AS-derived protein variants and circRNA translation products may be covered by noise or their cognate constitutive protein products in current proteomic studies. Further investigation into circRNA and AS necessitates novel technologies with higher resolution and sensitivity [211].

Recently, several online databases have been developed that focused on the expression, biological function and clinical implications of circRNAs in cancer (Table 2). While several platforms are not circRNA or cancer specific, they included circRNA and cancer as an important part of the whole repository [212, 213]. Notably, some of these platforms support online analysis and visualization with interactive and user-friendly web interfaces [214, 215]. With the development of single cell RNA sequencing techniques, researches are now able to summarize cell linage specific circRNA expression and explore their disease relevance [216]. Consulting these valuable resources will indeed bring great convenience, but the results should be further validated with thorough experiments and clinical trials before clinical translation.

**Table 2:** Online databases focused on circRNAs in cancer

Database	Main contents	URL	Reference
circExp	circRNA expression profile human cancers curated across 11 technical platforms	http://soft.bioinfominzhao.org/circexp/	[247]
CircNet 2.0	Integrative circRNA profile in human cancer exhibiting gene-circRNA-miRNA interaction network	https://awi.cuhk.edu.cn/∼CircNet	[248]
circMine	Disease specific circRNA database with online analytical and visualization function	http://hpcc.siat.ac.cn/circmine	[214]
CircR2Cancer	Manually curated and experimentally supported associations between circRNAs and cancers	http://www.biobdlab.cn:8000/	[249]
CRMarker	Manually curated repository of cancer circRNA markers with diagnostic and prognostic information	http://crmarker.hnnu.edu.cn/	[250]
CSCD2	Integrated interactional database of circRNA in human cancer with predicted miRNA-circRNA and RBP-circRNA interactions	http://geneyun.net/CSCD2	[215]
Lnc2Cancer 3.0	Experimentally supported lncRNA/circRNA cancer associations and web tools based on RNA-seq and scRNA-seq data	http://www.bio-bigdata.net/lnc2cancer	[251]
SCancerRNA	High confident experimentally supported circRNA-disease associations	http://www.scancerrna.com/BioMarker	[216]
SomamiR 2.0	Cancer somatic mutations altering microRNA-ceRNA interactions	http://compbio.uthsc.edu/SomamiR	[213]
MiOncoCirc	The first database to be composed primarily of circRNAs directly detected in tumor tissues	https://mioncocirc.github.io/	[9]
ncRNADrug	Validated and predicted ncRNAs associated with drug resistance and targeted by drugs	http://www.jianglab.cn/ncRNADrug	[212]

**Fig. 6:**
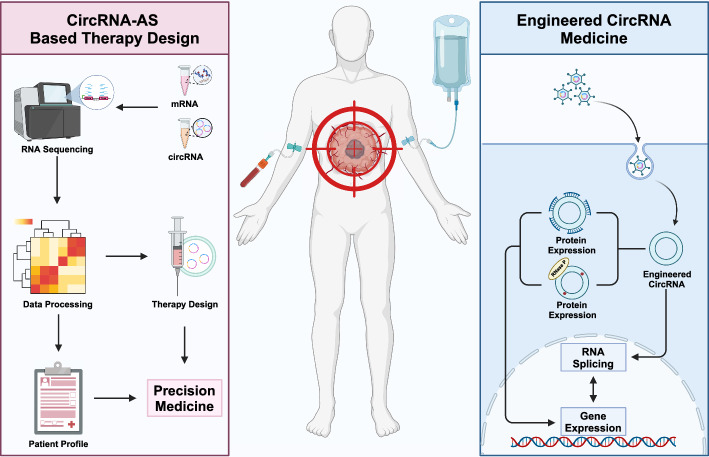
Therapeutic application of circRNA and alternative splicing. The crosstalk between circRNA and alternative splicing (AS) carries great therapeutic potential. Detecting alterations in patient circRNA transcriptome and AS pattern provides valuable information for molecular therapy design and a patient profile that is indicative of therapeutic response and prognosis, which contributes to precision medicine in cancer treatment. Moreover, circRNAs are promising therapeutic targets. Engineered circRNAs are capable of modulating gene expression through multiple mechanisms to modulate gene expression in cancer cells, thereby serving as a potent anticancer agent

AS and circRNAs provide valuable diagnostic and therapeutic clues for clinicians (Fig. 6). Detecting alterations in global splicing patterns and the occurrence of certain splicing variants may become a practicable diagnostic measure and predictive of prognosis and drug response. However, therapeutic attempts targeting AS should be inspected with caution. On the one hand, most factors, essentially speaking most RBPs, are versatile and participate in different steps of gene expression. On the other hand, even for splicing factors with clear functional relevance, such as QKI and ESRP1, target genes of the same splicing factor may impose opposite effects on cancers in a context-dependent manner. Therefore, simply abolishing or amplifying the activity of a splicing factor may not be a favorable therapeutic approach and accompanies imponderable side effects. Conversely, circRNAs are promising therapeutic targets or agents as well as valuable biomarkers for cancer diagnosis and prognostication owing to their innate stability. Engineered circRNAs have gained preliminary progress in vaccination, RNA-editing, and targeted protein expression [217–219]. Utilizing engineered antisense circRNA, researchers have successfully modulated exon skipping events to treat Duchenne muscular dystrophy in a mouse model [220]. Nevertheless, the complex crosstalk between AS and circRNA holds immense therapeutic potential. Resolving the crosstalk between AS and circRNA expands the potential of circRNA agents and helps bypass the possible side effects of aberrant AS events, paving the way for novel strategies to combat cancer.

## Data Availability

Not applicable.

## Abbreviations

ADAR: Adenosine deaminases acting on RNA
AML: Acute myeloid leukemia
AS: Alternative splicing
ceRNA: Competing endogenous RNA
circRNA: Circular RNA
ciRNA: Circular intronic RNA
CpG: Cytosine-phosphate-guanine
CTCF: CCCTC-binding factor
DHX9: DExH-box helicase 9
EIF4A3: Ekaryotic translation initiation factor 4A3
EMT: Epithelial-mesenchymal transition
EP300: E1A binding protein p300
ESRP1: Epithelial splicing regulatory protein 1
FUS: Fused in sarcoma
hnRNP: Heterogeneous nuclear ribonucleoprotein particle
ICS: Intronic complementary sequences
IRAlu element: Inverted repeated Alu element
IRES: Internal ribosome entry site
m^6^A modification: N^6^-methyladenosine modification
METTL3: Methyltransferase like 3
miRNA: MicroRNA
MRE: MicroRNA response element
NMD: Nonsense-mediated decay
pre-mRNA: Pre-messenger RNA
QKI: Quaking
snRNA: Small nuclear RNA
RBP: RNA binding protein
snRNP: Small nuclear ribonucleoprotein
SRSF1: Serine/arginine-rich splicing factor 1
TME: Tumor microenvironment
UTR: Untranslated region
YTHDF3: YTH N^6^-methyladenosine RNA binding protein F3
ZEB1: Zinc finger E-box binding homeobox 1
